# A Real-Time Defect Detection Strategy for Additive Manufacturing Processes Based on Deep Learning and Machine Vision Technologies

**DOI:** 10.3390/mi15010028

**Published:** 2023-12-22

**Authors:** Wei Wang, Peiren Wang, Hanzhong Zhang, Xiaoyi Chen, Guoqi Wang, Yang Lu, Min Chen, Haiyun Liu, Ji Li

**Affiliations:** 1 Key Laboratory of MEMS of the Ministry of Education, Southeast University, Nanjing 210096, China; 220204726@seu.edu.cn (W.W.); wang_peiren@seu.edu.cn (P.W.); zhanghz@seu.edu.cn (H.Z.); 220225906@seu.edu.cn (X.C.); wangguoqi_seu@aa.seu.edu.cn (G.W.); 220205700@seu.edu.cn (Y.L.); 2 School of Advanced Technology, Xi’an Jiaotong-Liverpool University, Suzhou 215400, China; min.chen@xjtlu.edu.cn; 3 College of Computer and Information, Hohai University, Nanjing 211100, China; haiyun_liu@hhu.edu.cn

**Keywords:** additive manufacturing, defect detection, deep learning, machine vision

## Abstract

Nowadays, additive manufacturing (AM) is advanced to deliver high-value end-use products rather than individual components. This evolution necessitates integrating multiple manufacturing processes to implement multi-material processing, much more complex structures, and the realization of end-user functionality. One significant product category that benefits from such advanced AM technologies is 3D microelectronics. However, the complexity of the entire manufacturing procedure and the various microstructures of 3D microelectronic products significantly intensified the risk of product failure due to fabrication defects. To respond to this challenge, this work presents a defect detection technology based on deep learning and machine vision for real-time monitoring of the AM fabrication process. We have proposed an enhanced YOLOv8 algorithm to train a defect detection model capable of identifying and evaluating defect images. To assess the feasibility of our approach, we took the extrusion 3D printing process as an application object and tailored a dataset comprising a total of 3550 images across four typical defect categories. Test results demonstrated that the improved YOLOv8 model achieved an impressive mean average precision (mAP50) of 91.7% at a frame rate of 71.9 frames per second.

## 1. Introduction

To date, additive manufacturing technologies have been significantly advanced to be able to develop 3D microelectronics due to their prominent advantages of design and fabrication flexibility, cost-effectiveness, and high customization [1,2,3,4]. Unfortunately, the process chain integrates multiple fabrication processes to deal with diverse materials and needs to build various micro-structures [5,6,7,8]. In this period, fabrication defects may occur due to diverse issues such as improper calibration, aiming errors, temperature fluctuation, inner stress, and material flow [9]. These defects significantly degrade the quality of electronic products and even result in total failure. Consequently, the development of in situ and non-destructive defect detection strategies for additive manufacturing technology has become an urgent task for ensuring the fabrication success of microelectronics. It can improve the quality and consistency of 3D printed parts by detecting and correcting errors in time and reduce material waste and time cost by avoiding the need to reprint defective parts or perform post-processing inspection. This method can further enable intelligent real-time feedback control for 3D printing by using machine learning to analyze the defect detected by the in-situ strategy.

The earliest detection method in additive manufacturing was artificial visual inspection. However, it faced challenges such as inefficiency, fatigue, and limited applicability. It fails to meet the efficiency and quality requirements of modern industrial production lines. Therefore, there is an urgent need to develop more efficient and reliable inspection technologies to address these limitations and enhance quality control in additive manufacturing processes [10,11]. 

ISO 9712 defines the non-destructive testing (NDT) method as a discipline that employs a physical principle [12]. Current NDT detection methods could be used for additive manufacturing processes including acoustic emission testing, eddy current testing, magnetic testing, and laser-ultrasonic testing, etc., which can precisely identify the location and the size of the defects [13,14,15]. However, these techniques normally rely on specific and costly equipment that is quite difficult to install within AM apparatus to realize in-situ inspection. 

Comparatively, machine vision has been regarded as a suitable candidate for in-situ and real-time detection [16,17,18,19]. Machine vision, as a measurement and inspection technology, improves detection efficiency, automation, real-time performance, and accuracy, particularly in large-scale and long-term industrial production processes. Machine vision technology only requires hardware for deploying the model and a camera for capturing images. Common defect detection methods based on machine vision include thresholding, edge detection, feature extraction, and description. Due to the development of deep learning, machine vision defect detection methods generally embrace neural networks and achieve real-time and intelligent working styles [16]. Moreover, these methods have also been applied in the AM area for defect detection. For example, G. Bakas et al. proposed a computer vision-based method for automatic defect detection in the fused deposition modelling (FDM) process [20]. Xu et al. developed an improved one-stage model based on the You Only Look Once (YOLO) v4 to detect the print quality of the FDM process [21]. However, both works only focused on the material filling defects and did not cover the major defects that often occurred in the extrusion-based AM processes, such as scratches, holes, and impurities, which largely limited the detecting capability of the systems.

Herein, we propose an advanced one-stage defect detection strategy based on machine vision and deep learning for AM processes, which enables high-precise in-situ and real-time detection of four defect categories in a variety of scenarios. Firstly, a defect dataset of an extrusion 3D printing is prepared via an industry camera. Secondly, the YOLOv8 neural network algorithm is improved by replacing the localization loss function and an attention mechanism into the backbone network, which significantly improves the detection capability of the model. Moreover, a defect quantification method is introduced to provide defect detection information depending on the number and the corresponding actual size of the image pixel. Finally, the proposed strategy is deployed and testified during the layer-by-layer extrusion 3D printing procedure.

## 2. Methods

### 2.1. 3D Printing

**Figure 1:**
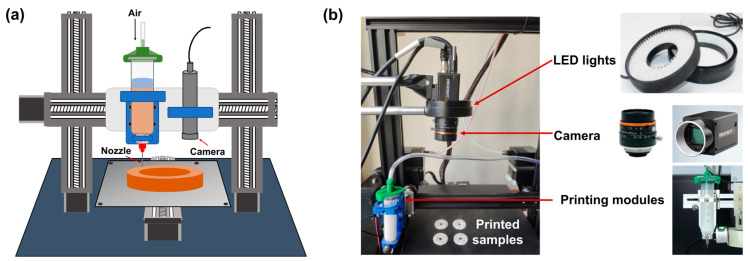
Extrusion 3D printing system with machine vision module. (**a**) The schematic diagram of the whole system. (**b**) The photos of the 3D printing system and image acquisition system.

All the samples were designed using AutoCAD 2019 software (AutoCAD, San Francisco, CA, USA) and saved in .stl format. Subsequently, the model was imported into slicing software and converted into G-code to operate a self-made extrusion-based 3D printer (Figure 1a) [22]. The printer’s core component consisted of a 3-axis CNC moving platform equipped with a precision pneumatic extruder. During the printing process, an air pump precisely controlled the extrusion of a calcium carbonate-based paste from the syringe nozzle. This paste was deposited layer-by-layer onto the substrate as guided by the CNC router’s movements. Key parameters for this work included a nozzle diameter of 510 μm, a layer height of 300 μm, an extrusion pressure of 40 kPa, and a nozzle moving speed of 250 mm/min.

### 2.2. Dataset Collection

**Figure 2:**
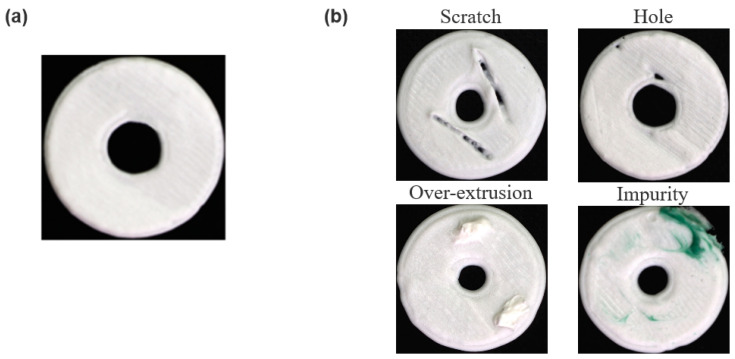
Photos of the front surface of the extrusion 3D printed layers. (**a**) The photo of the well-printed sample. (**b**) Four types of defects: scratch, hole, over-extrusion, and impurity.

To generate the defective dataset, we integrated an image acquisition system with a custom extrusion 3D printer to capture defect images (Figure 1b). In the extrusion 3D printing process, the morphology of each printed layer should be smooth without any cracks (Figure 2a). There are four primary defects including scratches, holes, over-extrusion, and impurities, that usually occur during printing (Figure 2b). These raw defect images underwent additional post-processing steps, including cropping, denoising, and annotation, to obtain high-resolution images devoid of background and noise interference.

#### 2.2.1. Defect Image Collection

Deep learning-based defect detection heavily depends on the quality of the datasets. Hence, we opted for a high-performance industrial camera, the Hikvision MV-CA-10GM/GC model, which offers a 6-megapixel resolution and utilizes Gigabit Ethernet (GigE) for swift and high-quality image transmission. The industrial camera is depicted in Figure 1b. The images of four defects including scratches, holes, over-extrusions, and impurities were captured, getting 3624 images in total.

#### 2.2.2. Image Check and Process

The captured images that were gathered underwent a manual inspection process, during which 74 images were identified as ineffective and subsequently eliminated from the dataset. These ineffective images were excluded due to reasons such as exposure abnormalities, the absence of discernible defects, or excessive defects that could not be adequately annotated. Ultimately, a dataset containing 3550 valid defect images for each defect type was obtained. The four defective categories are illustrated in Figure 2b.

The collected images required additional processing before they could be utilized as a training dataset. To eliminate irrelevant backgrounds that might interfere with training, we utilized Adobe Photoshop software 23.4.1 for image cropping, ensuring that the content of the printed product occupied more than 70% of each image. After cropping, 850 × 850 was the minimum size of all images in the dataset. To improve the model’s training efficiency and generalization ability, YOLOv8 would normalize the image size to 640 × 640 during model training. Therefore, all the images in the dataset can meet the resolution limit required for YOLOv8 model training. To reduce noise stemming from environmental factors during image acquisition, we applied Gaussian denoising on the image data and the Gaussian filter had a kernel size of 7 × 7 and a standard deviation of 1.5. For the annotation of the four defect categories, we employed the annotation software LabelImg 1.8.6, which recorded their locations and categories in corresponding .txt data files. Subsequently, the defect dataset was randomly divided into training, validation, and test sets. The training set comprised 80% of the images (2840 images), while both the validation and test sets contained 10% of the images (355 images each). This ratio was adopted by many previous works to ensure that the model has enough data to learn and avoid underfitting situations [23,24,25,26].

### 2.3. YOLOv8 Algorithm

To date, industrial defect detection predominantly relies on one-stage target detection algorithms from the you only look once (YOLO) series. YOLO has evolved rapidly since its inception in 2016. YOLO variants aim to better address the needs of object detection, including fast detection, high accuracy, and deployment on constrained edge devices. The latest version, YOLOv8 emphasizes real-time and high-classification performance while optimizing computational requirements. YOLOv8 has five models, namely YOLOv8 nano (YOLOv8n), YOLOv8 small (YOLOv8s), YOLOv8 medium (YOLOv8m), YOLOv8 large (YOLOv8l), and YOLOv8 extra large (YOLOv8x) depending on their model size. With the larger model, the accuracy is enhanced, while the speed is lower. Therefore, a balance between accuracy and speed needs to be made according to the requirements of specific applications. In this research, we employed YOLOv8n, the most recent iteration of the YOLO algorithm with the smallest model size to ensure high detection and diagnosis speed. By integrating an attention mechanism, enhancing the loss function, and devising a defect detection quantification method, we achieve intelligent defect detection in the AM process.

#### 2.3.1. Network Architecture

**Figure 3:**
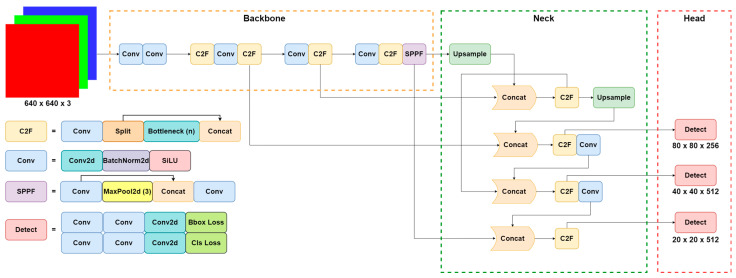
YOLOv8 network architecture.

Figure 3 illustrates the structure of YOLOv8, which consists of three main components: the backbone, neck, and head. The backbone serves as the fundamental framework of YOLOv8, comprised of a sequence of deep convolutional neural networks (CNN). These CNNs process the input image and extract high-level features. To achieve adaptive-sized output, the final layer of the backbone network employs spatial pyramid pooling fast (SPPF). The neck module utilizes a variant of the feature pyramid network (FPN). It combines features from various layers of the backbone network, merging them into a unified feature pyramid. This approach enables YOLOv8 to capture both low-level and high-level features concurrently, facilitating the detection of objects at different scales. The head module denotes the final component of YOLOv8′s architecture and is responsible for generating object detection predictions. YOLOv8 adopts an anchor-free model, eliminating the need for generating anchor boxes. This eliminates computational complexity while minimizing model storage requirements and computing resources.

#### 2.3.2. Attention Mechanism

**Figure 4:**
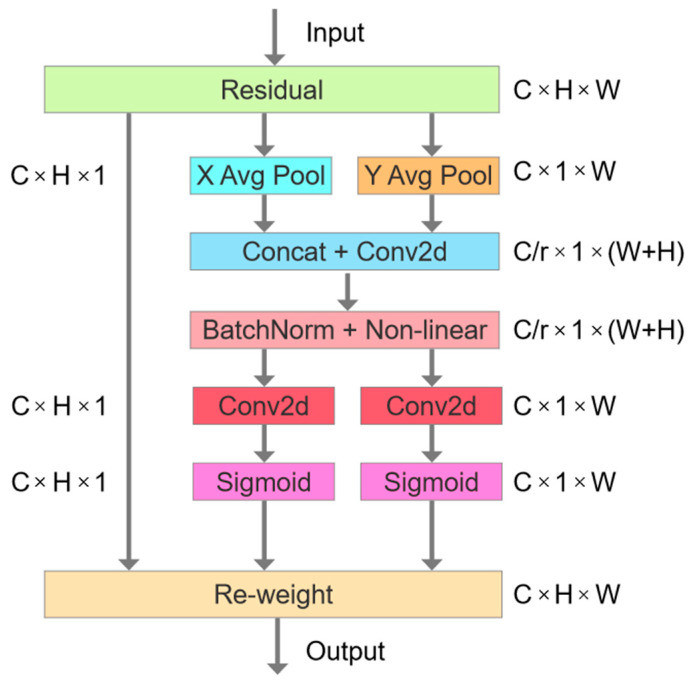
Coordinate attention (CA) structure.

When training the model, conventional YOLOv8 algorithms treat all the features as equally important, which can lead to inaccurate defect identification. To address this issue, an attention mechanism was added to enable networks to focus on crucial regions, thereby improving the accuracy and efficiency of defect detection. The bottleneck attention module (BAM) and convolutional block attention module (CBAM) extract positional attention information through convolutions after reducing the number of channels, but they struggle to capture long-range dependencies. In contrast, the recent coordinate attention (CA) method encodes both horizontal and vertical positional information, allowing networks to extract a wide range of spatial information without excessive computation. The structure diagram of CA is depicted in Figure 4 [27].

CA improves upon the capabilities of SE and CBAM by considering inter-channel and spatial information [28,29]. The process involves three steps: coordinate information embedding, spatial transformation, and weighted fusion. CA calculates a similarity matrix using position embedding vectors and performs weighted fusion with the feature map to get the final representation. The embedding method for horizontal and vertical coordinates is described as follows:

(1) $$z_{c}^{h}(h)=\frac{1}{W}\sum_{0\leq i<W}x_{c}(h,i)$$

(2) $$z_{c}^{w}(w)=\frac{1}{H}\sum_{0\leq j<H}x_{c}(j,w)$$

This method involves taking the input *x* and applying two pooling kernels with spatial extents of (*H*, 1) or (1, *W*) to encode each channel along the horizontal and vertical coordinates in the *c*-th channel. As a result, a pair of direction-aware feature maps is obtained.

**Figure 5:**
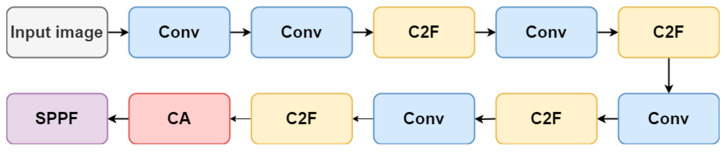
YOLOv8 improved the backbone network.

In this paper, the CA module is deployed in the preceding layer of the YOLOv8 backbone network’s SPPF module, which aims to enhance the algorithm’s feature learning capability and enable it to concentrate on relevant and valuable information. Figure 5 illustrates the structure of the backbone network following the CA module.

#### 2.3.3. EIOU Loss Function

In YOLOv8, the classification task utilizes the binary cross entropy (BCE) loss function, while the regression task combines the distribution focal loss (DFL) and complete intersection over union (CIOU) loss functions. Intersection over union (IOU) is a widely used evaluation metric in object detection, measuring the overlap between the predicted bounding box and the ground truth bounding box of an object. Both CIOU and efficient intersection over union (EIOU) are bounding box regression loss functions. The CIOU incorporates additional terms in its calculation to penalize variations in aspect ratios and adjusts the IOU value accordingly. However, it does not account for the orientation between the ground truth bounding box and the predicted box, leading to a slower convergence speed [30]. On the other hand, EIOU measures the similarity between the predicted box and the ground truth bounding box by considering the distances between their center point, width, and height. This enables better handling of scenarios involving rotation, occlusion, and misalignment among target boxes, ultimately improving detection accuracy [31]. The specific formula for the EIOU loss function is:

(3) $$\begin{array}{c} L_{EIOU}=L_{IOU}+L_{dis}+L_{asp}\\ \;=1-IOU+\frac{\rho^{2}\left(b,b^{gt}\right)}{{\left(w^{c}\right)}^{2}+{\left(h^{c}\right)}^{2}}+\frac{\rho^{2}\left(w,w^{gt}\right)}{{\left(w^{c}\right)}^{2}}+\frac{\rho^{2}\left(h,h^{gt}\right)}{{\left(h^{c}\right)}^{2}} \end{array}$$

$L_{IOU}$ denotes the *IOU* loss function; $L_{dis}$ is the center box distance loss function; $L_{asp}$ represents the length and width loss function; *IOU* denotes the intersection and union ratio, which is an indicator that measures the degree of overlap between the predicted box and the target box. For example, *w* denotes the width of the box, *h* is the height of the box, and *b* represents the center point of the box. w , $w^{gt}$ , and $w^{c}$ , respectively, denote the width of the predicted box, the width of the target box, and the width of the smallest enclosing box covering the two boxes.

EIOU is a more sensitive metric compared to CIOU since it considers the differences in width and height rather than solely relying on the aspect ratio. This characteristic allows EIOU to provide a more accurate measurement of the shape and size of bounding boxes, thereby improving the accuracy of object detection. Models that utilize the EIOU loss function demonstrate superior performance, particularly in detecting small and overlapping objects. In this paper, the EIOU loss function is adopted as an alternative to the CIOU loss function in model regression.

#### 2.3.4. Defect Information Evaluation

The detection algorithm mentioned above is capable of obtaining specific type and location information of defects, but it lacks the ability to provide detailed defect information such as defect size and area. To address this limitation, this paper introduces a method to extract defect sizes by analyzing the pixel information of the detected bounding boxes in the image. The proposed method establishes a proportional relationship between pixel sizes and physical sizes. The building platform of the 3D printer was used as the reference. The specific formula is:

(4) $$P=\frac{W}{w}$$

It measures the actual physical width of the printing platform (*W*) and the corresponding pixel width (*w*) in the obtained image. Then, we acquired the physical size (*P*) of each pixel in the image.

**Figure 6:**
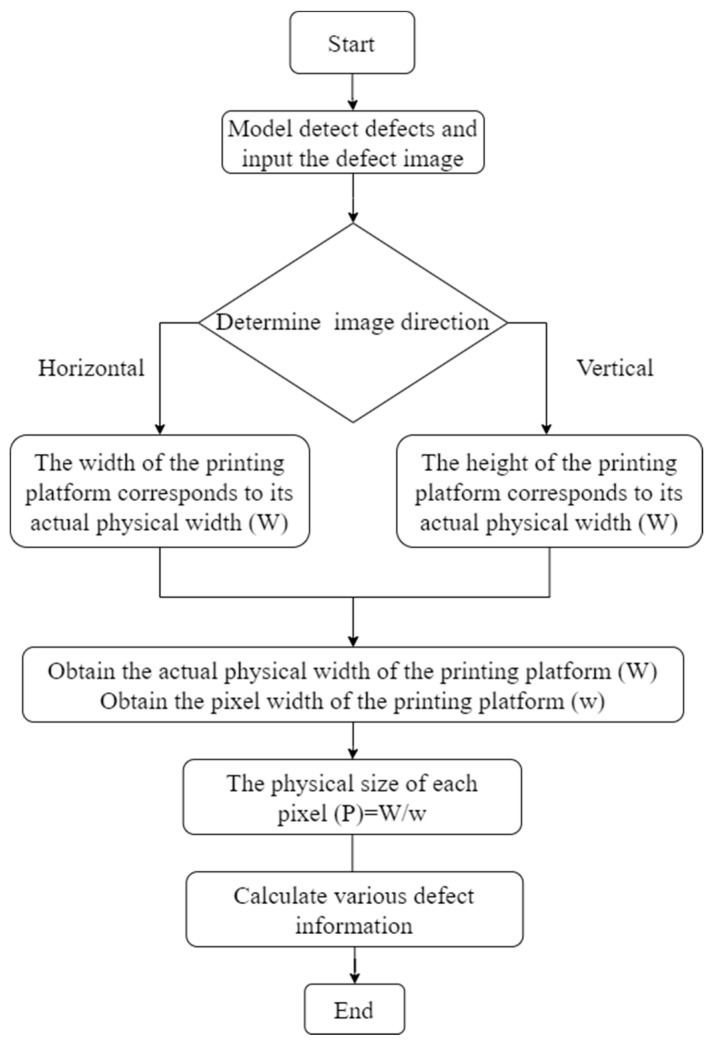
The flow chart of defect information evaluation.

The extraction of defect information involves several steps. First, when the defects are captured and identified, their corresponding image pixels are also obtained. Subsequently, based on the number and the actual size of the image pixels, the dimensions, area, and proportion of the defect area in the current printing layer can be calculated. If the area of the detection box exceeds one-fourth of the printed product modeling area, we define it as a special defect, indicating the severe defect is inspected on that layer. Finally, the diagnosis information of the total number of defects, the individual defects, and the defect area ratio are labeled in the detected image. The flow chart illustrating the process of defect information evaluation is presented in Figure 6.

## 3. Results

To validate the practicality of the proposed defect detection method, this paper chose the extrusion 3D printing process as the application object. The defective dataset acquired in Section 2.2.1 was used for model training. The performance of commonly used one-stage and two-stage target detection models was compared to show the superiority of YOLOv8n. The performance of improved YOLOv8n was further investigated via ablation experiments. The PyTorch framework was utilized for implementing the YOLOv8n defect detection model training [32]. The experimental setup included Python 3.8, CUDA 11.7, cuDNN 8.5, and an Nvidia GTX 3060Ti GPU with 8 GB memory, which provided the hardware infrastructure for training and running the model.

### 3.1. Evaluation Indicators

The performance of the detection model was evaluated using four key indicators: precision (P), recall (R), average precision (AP), mean average precision (mAP), and frames per second (FPS). These indicators provide comprehensive metrics to assess the accuracy and efficiency of the detection model [33]. The formulas of these indicators are as follows:

(5) $$Precision=\frac{TP}{TP+FP}$$

(6) $$Recall=\frac{TP}{TP+FN}$$

(7) $$AP=\int_{0}^{1}P(R)dR$$

During the evaluation of a test point, it can fall into one of the four categories: false positive (FP), the system incorrectly predicts the test point as positive when it is negative; false negative (FN), the system incorrectly predicts the test point as negative when it is positive; true positive (TP), the system correctly predicts the positive label for the test point; true negative (TN), the system correctly predicts the negative label for the test point.

Mean average precision (mAP) denotes the mean average precision of all classes, providing an overall performance measure for the model. The mAP formula is:

(8) $$mAP=\frac{1}{n}\sum_{i=1}^{n}AP_{i}$$

mAP50 is an indicator that measures the mean average precision across different classes when the IOU threshold is 50%. mAP50–95 is an indicator that measures the mean average precision across different classes by considering IOU thresholds ranging from 50% to 95%.

### 3.2. Performance Comparison Experiment

To evaluate the performance of YOLOv8n, we compared it with several state-of-the-art one-stage and two-stage object detection models, such as Faster R-CNN, Cascade R-CNN, YOLOv5s, YOLOv6n, YOLOv7, and PP-YOLOEs, based on our dataset. We tried to keep the training hyperparameters identical among these models during 300 training epochs. The specific data for each model’s performance testing are presented in Table 1.

According to the results, the original YOLOv8n model demonstrated the highest precision, recall, and mAP50 value among all the models evaluated. These results indicated that YOLOv8n provided better performance. In terms of speed, the two-stage models were normally slower compared to the one-stage models. This is because the two-stage models involve additional steps to generate candidate boxes, which increases computational requirements and complexity. YOLOv5s has the fastest model detection speed among all the models evaluated. This is because YOLOv5s uses the pruned CSP-Darknet53 network as a feature extractor, which reduces the feature map redundancy and improves the network parallelism, enhancing network efficiency and performance. Comparatively, other models like YOLOv6n use a deeper and wider network than YOLOv5s, which increases the computational cost and memory consumption. Considering both detection time and detection accuracy comprehensively, YOLOv8n was a better choice in these models.

### 3.3. Ablation Experiment

In this experiment, the network hyperparameters were configured as follows: the Adam optimizer was used for parameter tuning. The category confidence threshold for the target was set to 0.5, and the initial learning rate was 0.001. The WarmUp strategy was applied to adjust the initial learning rate. The batch size was set to 32 which meant that 32 images were processed in each training iteration, and the training process spanned 300 epochs, so the entire dataset was iterated 300 times during training. The input image size was normalized to 640 × 640, and 8 working threads were utilized.

In the experiment, the official YOLOv8n model was selected as the benchmark for model initialization. This model also has undergone transfer learning, which accelerates training and enhances performance for object detection tasks in new scenarios. Four models, named MDL 1–4, were trained with original YOLOv8n, YOLOv8n with CA, YOLOv8n with EIOU, and YOLOv8n with both CA and EIOU, respectively. The performance indices including precision, recall, average precision, and mean average precision were systematically compared and analyzed below.

**Figure 7:**
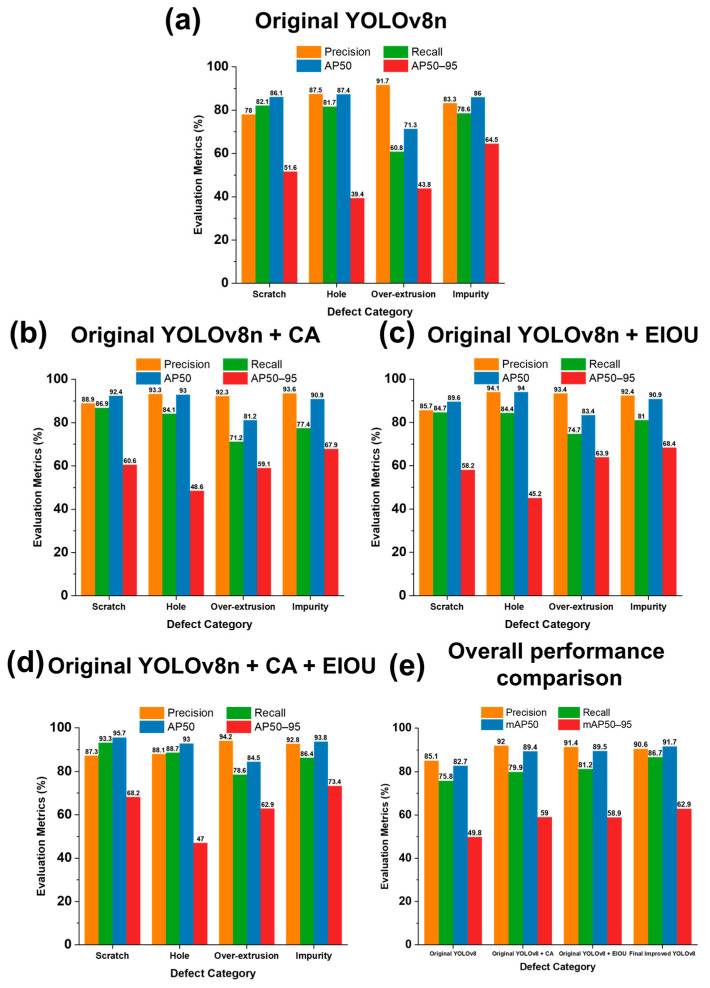
Detection performance of four YOLOv8n models.

The results of MDL 1, as shown in Figure 7a, exhibited a moderate level of detection performance. First, for the four defect categories, the precision, recall, and AP50 values were all around 80%. This indicated that the defect detection algorithm demonstrated competent capabilities in identifying different defect categories. Secondly, the AP50–95 values were considerably lower, mainly because the algorithm encountered challenges in achieving high accuracy in the higher IOU threshold range. Moreover, the over-extrusion defect category showed a higher precision but a lower recall rate. This phenomenon can be attributed to the fact that most small over-extrusion defects had little dissimilarity to the normal printing surface. Only when the over-extrusion material was severe, the protruding profile became distinguishable.

When the CA was incorporated into YOLOv8n training, the results of MDL 2 demonstrated a significant improvement in precision for all categories (Figure 7b). The precision of the defect categories of scratch and impurity has increased by more than 10%. This improvement is because CA made the model focus more on crucial areas during image processing, which avoids the interference of the image background. Accordingly, the attention mechanism enhances accuracy in target detection.

By utilizing the EIOU loss function, a remarkable enhancement of the overall recall rate was observed in Figure 7c. The EIOU loss function used could calculate the values of width and height independently rather than relying on their aspect ratio. This modification enables the rapid convergence of the model and provides superior performance in detecting small and overlapping objects.

Figure 7d illustrates the performance of MDL 4 which was trained by the YOLOv8n incorporating both CA and EIOU. This final model made a good balance between recall and precision. We summarized and compared the average performance of the four models for the four defect categories in Figure 7e. The values of the key indices were the average of those of the four types of defects. It was obvious that the MDL 4 achieved the highest mAP50 with a value of 91.7% compared to other models. The mAP50 comprehensively evaluates the precision and recall across multiple classes, which could be regarded as an important factor in assessing the accuracy of object detection. Therefore, the YOLOv8n model with both CA and EIOU significantly enhanced defect detection performance.

**Figure 8:**
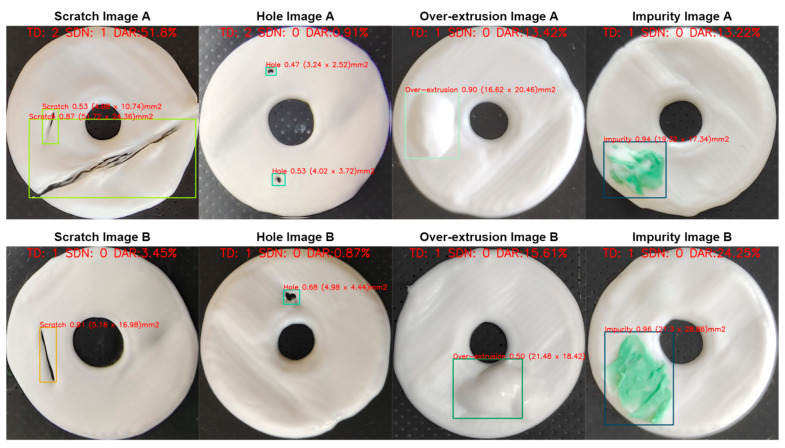
Defect detection images with labelled diagnosis information: total defects (TD), special defects number (SDN), defects area ratio (DAR).

**Figure 9:**
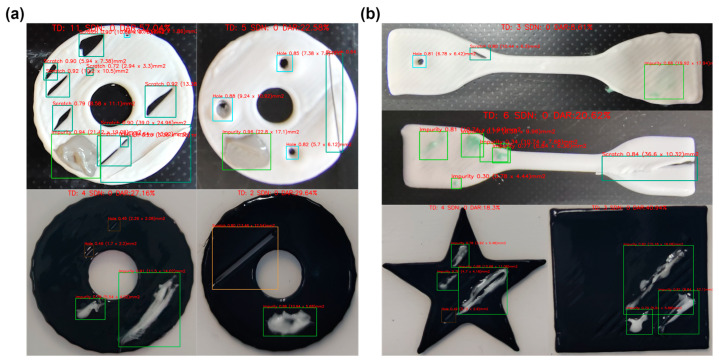
Defect detection images. (**a**) Images of multiple defects with the same shape. (**b**) Images of multiple defects with different shapes.

Figure 8 shows the detection and diagnosis results of the white disk-like samples with a single type of defect. The system successfully detected each defect in the samples. Figure 9a shows the detection and diagnosis results of the disk-like samples with different types of defects. For both white and black disks, the detection was correctly conducted, and different defects were identified simultaneously. For the samples with different shapes, our system also worked well distinguishing the different defects on the substrates with the shapes of dumbbell, star, and square (Figure 9b). Therefore, all these results showed that the proposed method can detect multiple defects in the samples with different backgrounds and structures.

### 3.4. System Integration

**Figure 10:**
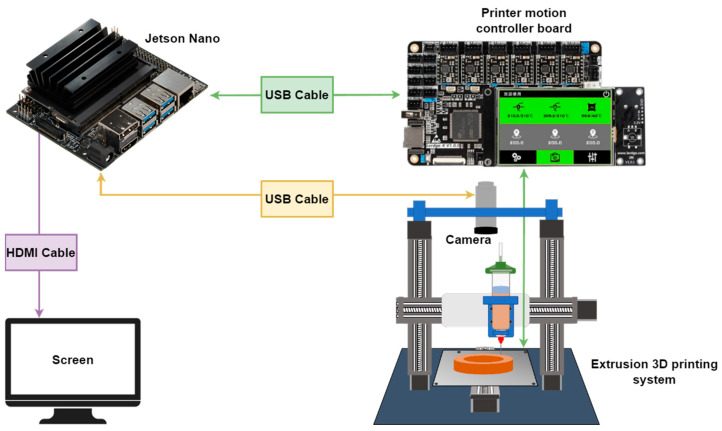
Jetson Nano-based integrated defect detection system for extrusion 3D printing apparatus.

According to Figure 10, the real-time defect detection module was integrated with the extrusion 3D printing apparatus using Jetson Nano as the platform. The Jetson Nano’s high-performance GPU efficiently accelerated the improved YOLOv8n model. Communication between the Jetson Nano and the 3D printer’s mainboard was established via USB. Additionally, Jetson Nano utilized a Python program to capture in-situ images with a camera. During the building of an object, when a layer printing is completed, the nozzle returns to the origin, and the camera is triggered to capture the image of this layer. Subsequently, the printing is restarted, and simultaneously the previously captured image is processed by the improved YOLOv8n model for detection and diagnosis. After the whole printing is completed, a comprehensive diagnostic report of the entire sample can be obtained based on the analysis information of each layer. In the future, a real-time feedback control mechanism can be developed to adjust the printing parameters on time based on the diagnostic results of each layer, which will significantly enhance the fabrication quality of additive manufacturing technologies.

## 4. Conclusions

In this study, we proposed an in situ defect detection strategy that integrates machine vision and deep learning technologies into additive manufacturing processes. An improved YOLOv8 algorithm with coordinate attention (CA) and EIOU loss function was developed to train the defect detection model with a self-made defect image dataset. This model could be employed to monitor fabrication defects in situ during the 3D printing procedure. The typical defects of the extrusion 3D printing process were prepared in the dataset to testify to the performance of this strategy. Experimental results showed that the improved YOLOv8n model achieves a mean average precision (mAP50) of 91.7% at a high speed of 71.9 FPS. We have deployed the proposed strategy to an extrusion 3D printing apparatus for real-time quality monitoring. However, this method still has two main limitations: (1) the defect depth information cannot be reflected in the captured 2D images, so it is difficult to obtain three-dimensional information of the defects in the additively manufactured products; (2) The improved YOLOv8 used in this work is a supervised algorithm which needs to label a large number of datasets during data annotation, and this step usually consumed a lot of labour and time. In the future, 3D printing settings can be adjusted depending on the feedback of the defect detection module in time to ensure a high success rate in additive manufacturing.

## Figures and Tables

**Table 1:** Detailed performance of each detection model.

Scheme	Precision	Recall	mAP50	FPS
Faster R-CNN	76.6%	68.8%	74.5%	5
Cascade R-CNN	75.1%	75.2%	78.4%	4
YOLOv5s	77.8%	68.0%	74.2%	**100**
YOLOv6n	80.8%	73.1%	79.1%	45
YOLOv7	80.1%	68.5%	76.7%	83
PP-YOLOEs	80.2%	74.3%	78.8%	66
YOLOv8n	**85.1%**	**75.8%**	**82.7%**	90

## Data Availability

Data are contained within the article.
